# Anticipatory pleasure predicts effective connectivity in the mesolimbic system

**DOI:** 10.3389/fnbeh.2015.00217

**Published:** 2015-08-12

**Authors:** Zhi Li, Chao Yan, Wei-Zhen Xie, Ke Li, Ya-Wei Zeng, Zhen Jin, Eric F. C. Cheung, Raymond C. K. Chan

**Affiliations:** ^1^Neuropsychology and Applied Cognitive Neuroscience Laboratory, Key Laboratory of Mental Health, Institute of Psychology, Chinese Academy of SciencesBeijing, China; ^2^The University of Chinese Academy of SciencesBeijing, China; ^3^Key Laboratory of Brain Functional Genomics (MOE and STCSM), School of Psychology and Cognitive Science, East China Normal UniversityShanghai, China; ^4^Department of Psychology, University of CaliforniaRiverside, CA, USA; ^5^MRI Center, Hospital 306 of PLABeijing, China; ^6^Castle Peak Hospital, Hong Kong Special Administrative RegionTuen Mun, China

**Keywords:** hedonic capacity, monetary reward, dynamic causal modeling, anticipatory pleasure

## Abstract

Convergent evidence suggests the important role of the mesolimbic pathway in anticipating monetary rewards. However, the underlying mechanism of how the sub-regions interact with each other is still not clearly understood. Using dynamic causal modeling, we constructed a reward-related network for anticipating monetary reward using the Monetary Incentive Delay Task. Twenty-six healthy adolescents (Female/Male = 11/15; age = 18.69 ± 1.35 years; education = 12 ± 1.58 years) participated in the present study. The best-fit network involved the right substantia nigra/ventral tegmental area (SN/VTA), the right nucleus accumbens (NAcc) and the right thalamus, which were all activated during anticipation of monetary gain and loss. The SN/VTA directly activates the NAcc and the thalamus. More importantly, monetary gain modulated the connectivity from the SN/VTA to the NAcc and this was significantly correlated with subjective anticipatory pleasure (*r* = 0.649, *p* < 0.001). Our findings suggest that activity in the mesolimbic pathway during the anticipation of monetary reward could to some extent be predicted by subjective anticipatory pleasure.

## Introduction

Deficits in hedonic capacity, namely anhedonia, are often found in patients with schizophrenia, bipolar disorder, major depression, substance addiction, anxiety, and eating disorders (Shankman et al., 2014). Traditional symptom-based psychiatric diagnosis may not be able to capture these underlying features that cut across diagnostic entities. The recently proposed Research Domain Criteria (RDoC) aims to address this problem (Cuthbert, 2014; Cuthbert and Workgrp, 2014). The RDoC suggests researchers to focus on elemental cognitive and emotional functions, such as hedonic capacity, using various approaches ranging from behavioral performance, through brain circuits, to genes (NIMH, 2008). The Monetary Incentive Delay (MID) task (Knutson et al., 2000) and the Temporal Experience of Pleasure Scale (TEPS; Gard et al., 2006) have been suggested as appropriate instruments to examine the two components of hedonic experience, namely anticipatory and consummatory pleasure. Converging evidence suggests that the nucleus accumbens (NAcc) is a vital hedonic hotspot in anticipatory pleasure (Kringelbach and Berridge, 2009; Berridge and Kringelbach, 2013). Using the MID task, activation of the NAcc has been observed during anticipation of secondary rewards (Knutson et al., 2000, 2001). Similar results have been reported in anticipation of primary rewards, such as sucrose solution (O’Doherty et al., 2002) and social rewards (Kohls et al., 2013). The NAcc appears to play a key role in integrating information from the midbrain, the limbic system and the frontal cortex to facilitate appropriate choice and goal-directed behavior (Camara et al., 2009). The substantia nigra/ventral tegmental area (SN/VTA) also plays an important role in reward processing (Horvitz, 2000; Duezel et al., 2009). Anticipation of primary and secondary rewards, taste (O’Doherty et al., 2002), money (Breiter et al., 2001), and happy faces (Aharon et al., 2001) activates the SN/VTA. Lastly, the thalamus also plays a role in hedonic experience. Previous studies have reported activation of the thalamus in anticipation of rewards (Knutson et al., 2000, 2001; Knutson and Greer, 2008). The thalamus integrates messages from the emotional, cognitive, and motor cortices and relays information to the frontal cortex to formulate goal-directed behavior (Haber and Calzavara, 2009). In addition, the thalamus also appears to be important in retrospective and prospective coding for predicted reward (Komura et al., 2001). The NAcc-nigra-thalamic circuit is involved in the regulatory function of the thalamus in reward processing (Montaron et al., 1996). Our study (Chan et al., in press), adopting the modified MID task, showed that anticipation of monetary gain activated the NAcc, the globus pallidus and the thalamus, whereas anticipation of monetary loss activated the NAcc, the thalamus and the SN/VTA. These findings further support the important roles of the SN/VTA, the NAcc and the thalamus during anticipation of rewards.

**FIGURE 1:**
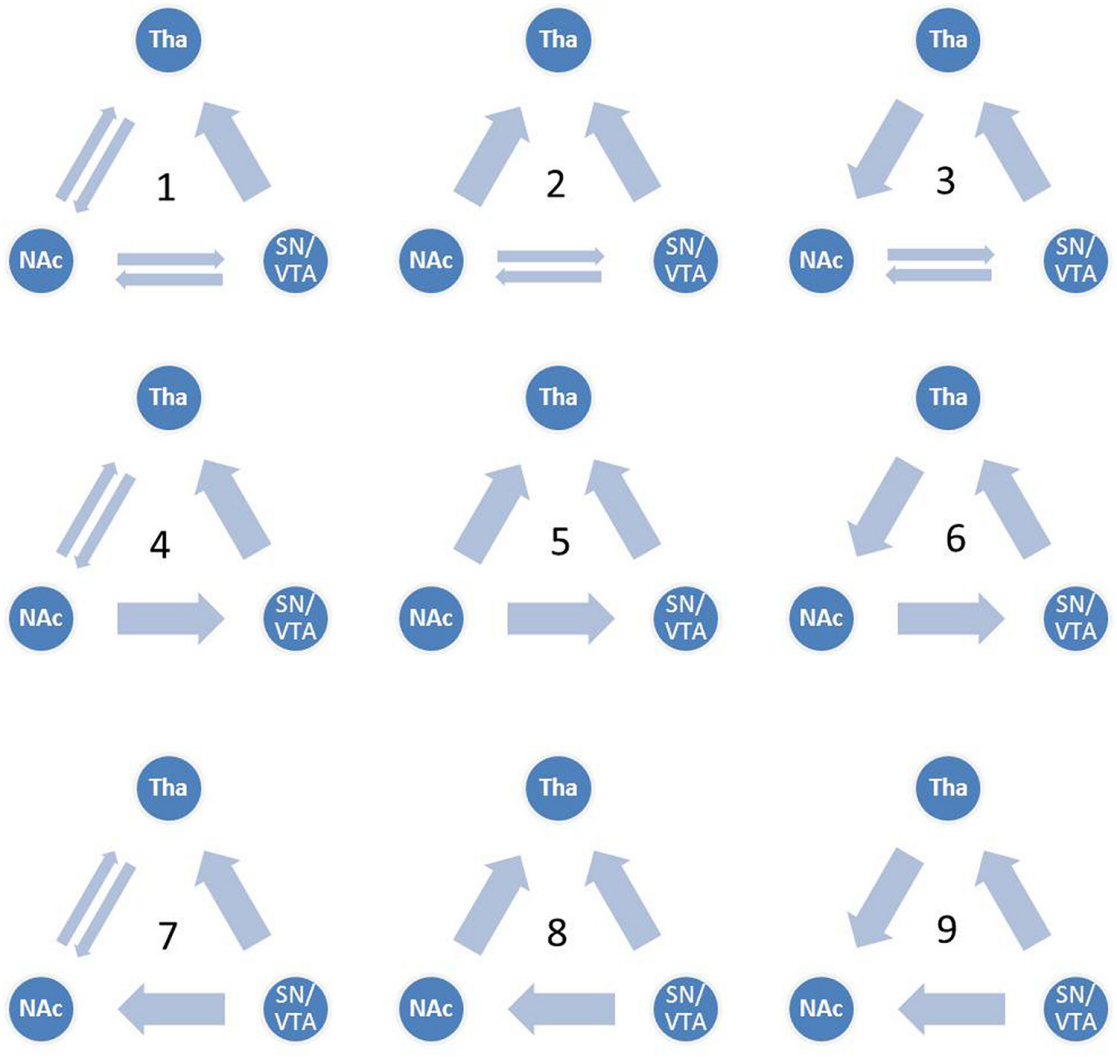
**Candidate dynamic causal models.** All nine candidate models are shown. Model 1 contains reciprocal pathways between the substantia nigra/ventral tegmental area (SN/VTA) and the nucleus accumbens (NAcc), reciprocal pathways between the NAcc and the thalamus and a non-reciprocal pathway from the SN/VTA to the thalamus. External inputs, including anticipation of monetary gain and loss, initially impact the SN/VTA, followed by the NAcc and the thalamus.

Although the functional connectivity between the NAcc, the SN/VTA and the thalamus have been a focus of recent studies in hedonic capacity (Camara et al., 2009; Haber and Calzavara, 2009; Cauda et al., 2011), to the best of our knowledge, few studies had examined the relationships between these three regions, especially the interaction between the SN/VTA and the NAcc during anticipation of rewards. Some studies have used dynamic causal modeling (DCM) to investigate reward related circuits (Alexander and Brown, 2010; Veldhuizen et al., 2011; Gonen et al., 2012; Cho et al., 2013; Yu et al., 2013). Using DCM, Veldhuizen et al. (2011) found that the anterior insular represents breaches of taste identity by receiving afferent connectivity from the ventral striatum and the inferior parietal cortex. Furthermore, by reciprocal connectivity from the amygdala, the ventral striatum plays a role in anticipating the attractability of human faces (Yu et al., 2013). Gonen et al. (2012) found that the VTA and the NAcc are related to the behavioral activation system and the NAcc represents the reward by cooperating with the dorsal medial prefrontal cortex. In addition, brain activity in the substantia nigra was found to be capable of predicting dopamine release in the NAcc during the anticipation of rewards (Schott et al., 2008). These findings suggest the vital role of the VS, especially the NAcc, in the reward circuit. In addition, the SN/VTA may also be engaged during the anticipation of rewards. However, the causal relationship between the SN/VTA and the NAcc is still unknown. In the present study, we constructed nine dynamic causal models between the SN/VTA, the NAcc and the thalamus, which contained reciprocal pathways between the SN/VTA and the NAcc, and between the NAcc and the thalamus and a non-reciprocal pathway from the SN/VTA to the thalamus (**Figure 1**). Taking into account the complexity of the models, the connectivity from the thalamus to the SN/VTA was excluded from consideration because this anatomical connectivity is not as clear-cut as the other five. Moreover, we measured the subjective anticipatory and consummatory pleasure of participants and correlated them with the parameters of the best-fit model.

Given these and our previous work using the MID task, we examined the reward-related network for anticipating monetary reward. We hypothesized that (1) the NAcc, the SN/VTA and the thalamus would all be activated during anticipation of monetary gain and loss; (2) the SN/VTA would exert a direct effect on the NAcc whereas the thalamus would integrate information from the SN/VTA and the NAcc.

## Materials and Methods

### Participants

Twenty-six (11 females) healthy right-handed adolescents with a mean age of 18.6 years (sd = 1.35), a mean duration of education of 12 years (sd = 1.58) and a mean IQ estimate of 95.38 (sd = 13.56) [estimated by the shot-form of the Chinese version of the Wechsler Adult Intelligence Scale-Revised (WAIS-R; Gong, 1992)] were recruited from the community. Exclusion criteria included: a personal or family history of mental illness, a history of head injury, and a history of substance abuse. The study was approved by the Ethics Committee of the Institute of Psychology, the Chinese Academy of Sciences. Written informed consents were obtained from all participants. Each participant was recompensed with 100 CNY (China yuan) and the monetary rewards they acquired in the MID task after the completion of the study.

### Temporal Experience Pleasure Scale

The TEPS was designed to measure anticipatory and consummatory pleasure (Gard et al., 2006). We used the Chinese version of the TEPS which consists of 10 items with a six-point Likert scale measuring anticipatory pleasure and 10 items measuring consummatory pleasure (Chan et al., 2012). The items measuring anticipatory pleasure capture the pleasure experienced during the anticipation of positive events, such as “When I hear about a new movie starring my favorite actor, I can’t wait to see it,” whereas the items measuring consummatory pleasure capture the pleasure experienced during the consummation of positive events, such as “A hot cup of coffee or tea on a cold morning is very satisfying to me.” Higher score on the TEPS indicates higher hedonic capacity. Both the original and the Chinese version of the TEPS have been shown to have satisfactory reliability and validity (Gard et al., 2006; Chan et al., 2012).

### Monetary Incentive Delay Task (MID)

In this study, we used an abbreviated version of the MID task developed by Chan et al. (in press). A cue lasting 250 ms was presented on a projection screen which was reflected in a small mirror fixed on the head coil of the scanner, followed by the first interval and then a blue cross that the participants were asked to quickly hit by pressing a button. Then the second interval was presented which was followed by the monetary stimuli. The cues consisted of a triangle signifying the gain condition, a square signifying the loss condition and a circle signifying the neutral condition. In the gain condition, participants could gain five monetary points if the blue cross was hit. In the loss condition, participants would lose five monetary points if the blue cross was not hit. In the neutral condition, participants would gain or lose nothing whether the blue cross was hit or not. The duration of intervals were randomized to avoid participants from anticipating the blue cross and to maintain the duration of each trial at 12 s. In addition, the duration of the blue cross was jittered around 300 ms according to the performance of each participant to maintain the accuracy at about 66%. Participants were asked to perform two runs of the task. Each run contained 30 trials, 10 gain conditions, 10 loss conditions, 10 neutral conditions, and a blank screen lasting for 8 s presented in the first instance for a dummy scan. The order of the trials was pseudorandom across participants and different between the two runs. Participants were told that the final remuneration would be 100 CNY plus the monetary points they obtained in the task. The average hit rate, the reaction time by condition and the earnings are presented in Supplementary Table S1.

### Functional MRI Data Collection

Imaging data were collected in a Siemens 3T Trio scanner with a 32-channel head coil at the MRI Centre of the 306 Hospital in Beijing. T2^∗^-weighted echo planner ingredient sequence (TR = 2000 ms; TE = 30 ms; FOV = 210 mm; slices = 32; flip angle = 90°; image matrix = 64 × 64; voxel dimensions = 3.3 mm × 3.3 mm × 4 mm) was applied to acquire functional brain images. Then a high resolution T1 structure image was obtained with the sequence: TR = 2300 ms; TE = 3 ms; FOV = 256 mm; flip angle = 9°; image matrix = 256 × 256; voxel dimensions = 1 mm × 1 mm × 1 mm.

### Functional MRI Data Processing

All the fMRI data were analyzed with the free software Statistical Parameter Mapping 8 (SPM8, Wellcome Trust Centre for Neuroimaging, London, UK). Before pre-processing, the first four dummy scans were discarded. After slice timing correction, images were realigned to the twentieth slice of each TR. Then the mean EPI image was normalized to the single person template of the Montreal Neurological Institute. Finally all the images were smoothed with a Gaussian kernel of 5 mm full-width half-maximum.

Based on the canonical haemodynamic response function (HRF), only the three anticipatory events: the monetary gain, the monetary loss, and the neutral condition, were included in the general linear modeling. Besides, the six parameters of head movement generated in the realignment were included in the modeling as covariates. The contrast ‘gain – neutral’ was designed to examine brain activities in response to monetary gain, and the contrast ‘loss – neutral’ was designed to examine brain activities in response to monetary loss. The contrast ‘all – neutral’ referred to the general effect of monetary stimuli and was used in the DCM. The contrasts of each participant were included in a one-sample *t*-test which was set in the second-level analysis of the SPM8.

Since we aimed to examine the function of the SN/VTA, the NAcc, and the thalamus and their interaction during the anticipation of monetary stimuli, we analyzed brain activation with pre-defined regions of interest (ROI). The ROIs of the NAcc and the thalamus were selected from the Harvard-Oxford subcortical structure atlas. The ROI of the SN/VTA was adopted from a very high resolution subcortical probabilistic atlas which was quantified with a 7T structure MRI (Keuken et al., 2014). The three ROIs, were masked on the contrast ‘gain – neutral’ and the contrast ‘loss – neutral’ respectively. Small volume correction (SVC) within an 8-mm radius sphere was applied. The statistical threshold was set as familiar-wise-error (FWE) correction with *p* < 0.05.

### Dynamic Causal Modeling

Before the procedure, the time courses of the SN/VTA, the NAcc, and the thalamus were extracted from the contrast ‘all – neutral’ of all participants. The ROIs were defined as the overlaps between the masks used in the ROI analysis with an 8-mm radius sphere centered around the peak points activated in the SN/VTA, the NAcc, and the thalamus, respectively. The statistical threshold was set as uncorrected *p* < 0.05.

We constructed nine dynamic causal models. The complete model, Model 1, contained reciprocal connectivity between the SN/VTA and the NAcc, reciprocal connectivity between the NAcc and the thalamus, and a non-reciprocal connectivity from the SN/VTA to the thalamus. From Model 1, one or two connectivity was subtracted in different ways to form eight other models (**Figure 1**). The right SN/VTA, the right NAcc, and the right thalamus were included in the model for their stronger activations than their left- sided counterparts (**Table 1**). Using the SPM8, a HRF was constructed, which contained the event ‘all,’ ‘gain,’ and ‘loss’. The event ‘all’ was defined as an input at the SN/VTA which is axiomatically considered a dopamine-rich region projecting to the terminals of the NAcc (Duezel et al., 2009; Haber and Knutson, 2010; Cauda et al., 2011). For the exploratory aim of this study and the unclear effect of valence to the connectivity of the reward circuit, the event ‘gain’ and ‘loss’ were, respectively, defined as perturbations to all the intrinsic connectivity, or the edges, of the models. A random-effect analysis of Bayesian Model Selection (BMS) was applied to identify the best-fit model with the highest exceedance probability (Friston et al., 2003; Stephan et al., 2010). Then the endogenous and perturbed parameters of the best-fit model were imported into the Predictive Analytics Software 18.0 (PASW 18.0) for significance testing. Bonferroni correction was applied to correct for multiple comparison. Finally, all the parameters were correlated with the total, the anticipatory and the consummatory subscale scores of the TEPS using Pearson Correlation.

**Table 1:** Analysis of regions of interest (ROI) during the anticipation to monetary rewards.

Contrasts and areas	Side	*p*-value	Peak T	Coorinate (x,y,z)
**All cues > Neutral cues**			
Nucleus Accumbens (NAcc)	Right	<0.0001	10.46	6,6,-3
NAcc	Left	<0.0001	9.74	-6,6,-3
Substantia nigra/ventral tegmental area (SN/VTA)	Right	<0.0001	9.29	12,-24,-15
SN/VTA	Left	<0.0001	9.03	-9,-24,-15
Thalamus	Right	<0.0001	9.2	6,-3,-3
Thalamus	Left	<0.0001	9.18	-6,-3,-3
**Gain cues > Neutral cues**			
NAcc	Right	<0.0001	8.77	6,9,-3
NAcc	Left	<0.0001	8.35	-6,6,-6
SN/VTA	Right	<0.0001	8.62	9,-24,-15
SN/VTA	Left	<0.0001	8.35	-9,-24,-15
Thalamus	Right	<0.0001	8.25	6,-15,9
Thalamus	Left	<0.0001	8.81	-12,-18,15
**Loss cues > Neutral cues**			
NAcc	Right	<0.0001	11.67	6,6,-3
NAcc	Left	<0.0001	10.57	-6,6,-3
SN/VTA	Right	<0.0001	9.01	12,-21,-15
SN/VTA	Left	<0.0001	8.08	-9,-24,-15
Thalamus	Right	<0.0001	10.31	6,-3,-3
Thalamus	Left	<0.0001	9.34	-3,-9,6

*The threshold was set as p < 0.05 with family-wise error (FWE) correction, small volume correction (SVC)*.

## Results

### Regions of Interest Analysis

The bilateral NAcc, the bilateral SN/VTA and the bilateral thalamus were all significantly activated to the contrasts ‘gain – neutral,’ ‘loss – neutral,’ and ‘all – neutral’ (**Table 1**).

### Dynamic Causal Modeling

**FIGURE 2:**
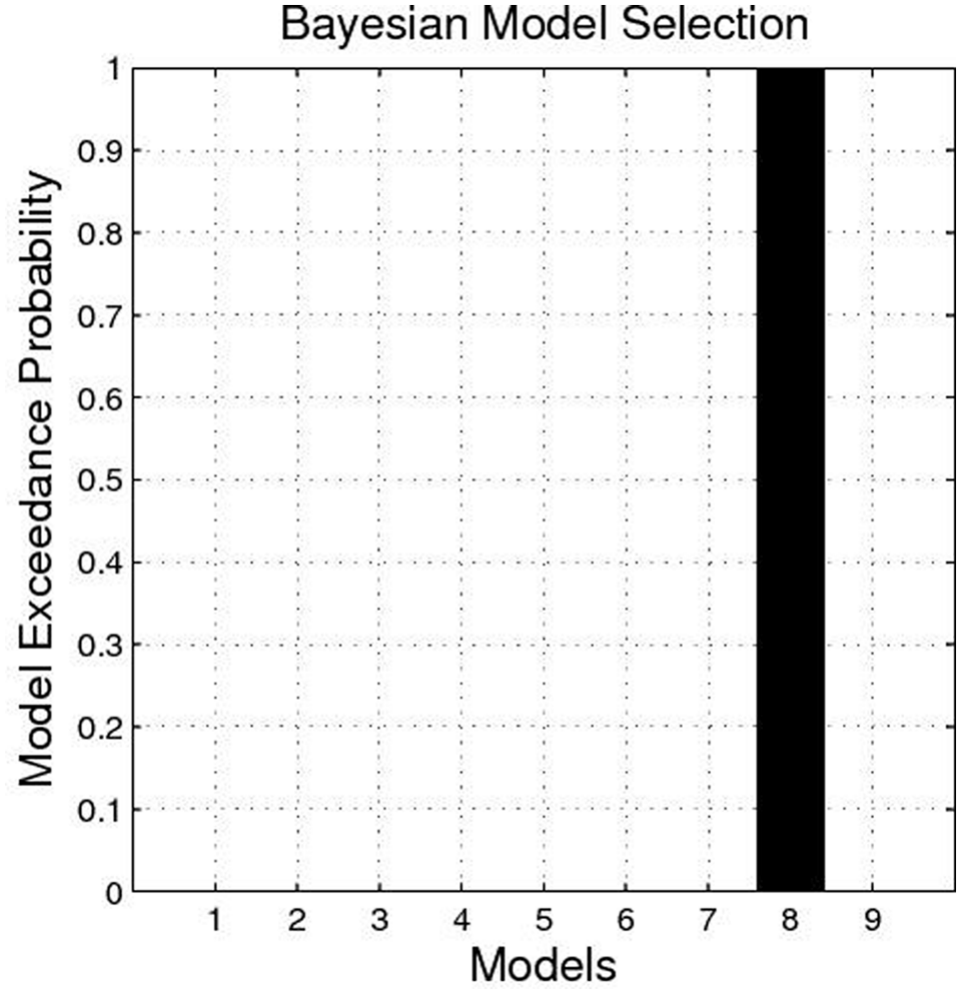
**Exceedance probability of each model.** The exceedance probability of Model 8 is higher than the other eight models.

**FIGURE 3:**
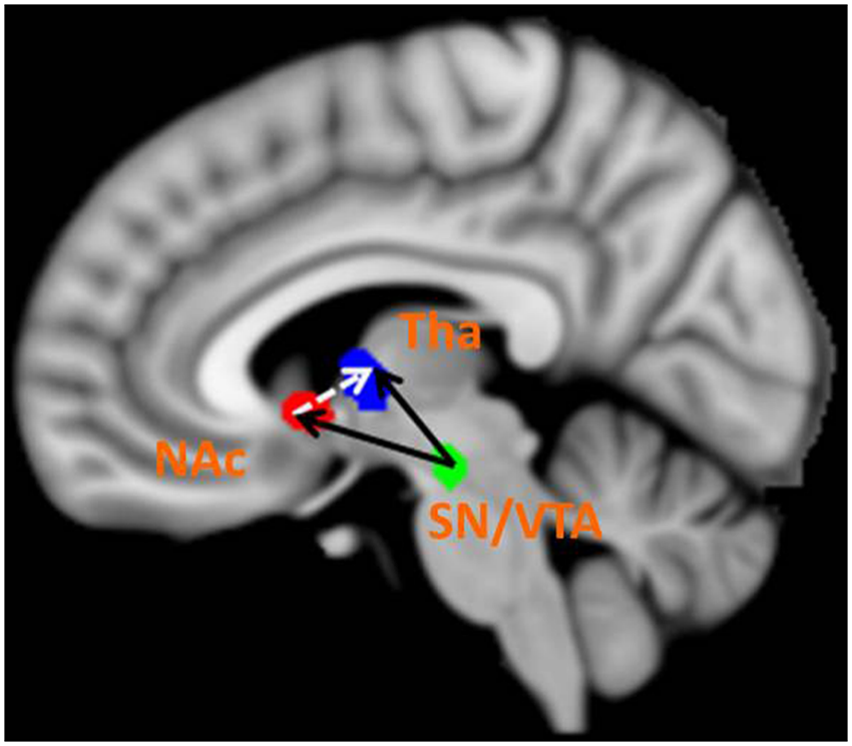
**Best-fit dynamic causal model.** Model 8 is the best model in fitting the time series of the SN/VTA, the NAcc, and the thalamus. In Model 8, the SN/VTA projects two causal pathways to the NAcc and the thalamus, whereas the NAcc exerts a causal influence on the thalamus.

The BMS identified Model 8 as the best-fit model with the highest exceedance probability during the anticipation of monetary stimuli (**Figures 2** and **3**). The endogenous connectivity of Model 8 contained two causal pathways from the SN/VTA to the NAcc and to the thalamus, while the NAcc had a causal effect on the thalamus. Endogenous parameters of the pathway from the SN/VTA to the NAcc [*t*(25) = 10.96, *p* < 0.001] and to the thalamus [*t*(25) = 8.42, *p* < 0.001] were significant, while that from the NAcc to the thalamus was not [*t*(25) = 1.27, *p* = 0.217] (**Table 2**).

**Table 2:** Parameters of endogenous connections and modulation of the Model 8.

Path	Endogenous Connection	Modulation_gain	Modulation_loss
		Mean ± SEM	Mean ± SEM
SN/VTA→NAc	1.24 ± 0.15^∗∗^	0.55 ± 0.08^∗∗^	0.19 ± 0.04^∗∗^
SN/VTA→Tha	1.96 ± 0.18^∗∗^	0.28 ± 0.03^∗∗^	0.17 ± 0.03^∗∗^
NAc→Tha	0.09 ± 0.07	-0.26 ± 0.03^∗∗^	-0.20 ± 0.03^∗∗^

*SN/VTA, Substantia nigra/ventral tegmental area; Tha, Thalamus; NAc, Nucleus accumbens; ^∗∗^p < 0.01.*

As for the modulation parameters caused by external experimental stimuli in the Model 8, both monetary ‘gain’ and ‘loss’ modulated the connectivity from the SN/VTA to the NAcc, from the SN/VTA to the thalamus and from the NAcc to the thalamus (**Table 2**).

### Correlation between Subjective Pleasure Experience and Modeling Parameters

**FIGURE 4:**
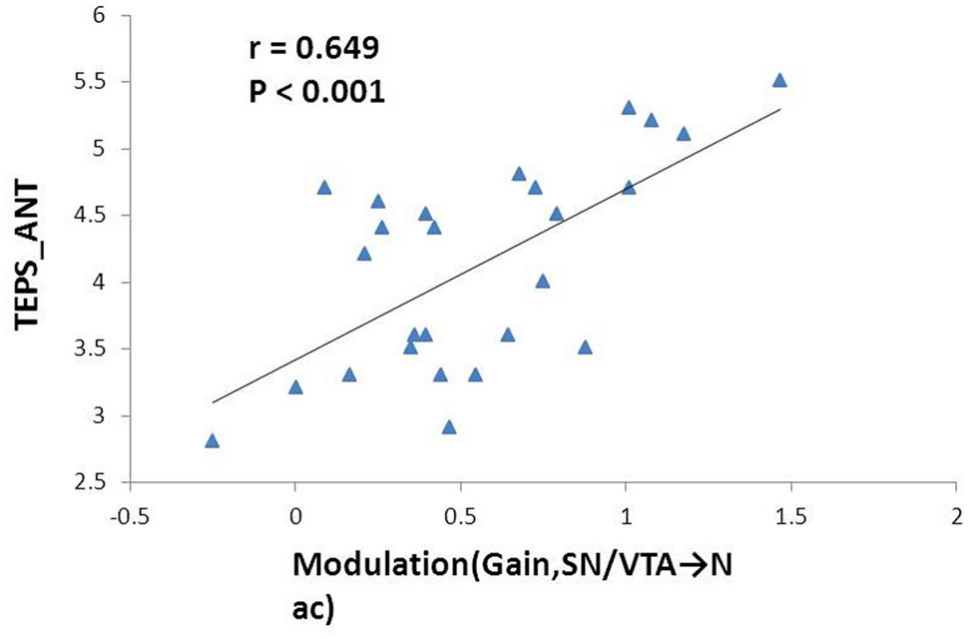
**Correlation between the effect of monetary gain on the mesolimbic pathway and anticipatory pleasure experience.** Modulation of monetary gain on the causal connectivity from the SN/VTA to the NAcc positively correlates with anticipatory pleasure experience. The Pearson correlation coefficient remains significant after Bonferroni correction.

The mean total TEPS score of the participants was 78.18 ± 12.72, while the mean anticipatory and consummatory subscale scores were 41.78 ± 6.9 and 36.4 ± 7.39, respectively. We found significant positive correlation between the modulation by ‘gain’ events on the nigrostriatal pathway from the SN/VTA to the NAcc with anticipatory subscale score (*r* = 0.649, *p* < 0.001) and total score on the TEPS (*r* = 0.555, *p* = 0.003). The former correlation remained significant after Bonferroni correction (**Figure 4**).

## Discussion

In the present study, using the MID Task, we observed activation of the SN/VTA, the NAcc, and the thalamus in healthy adolescents during anticipation of both monetary gain and loss. We found that the SN/VTA projects two causal pathways to the NAcc and the thalamus. Importantly, the causal connection from the SN/VTA to the NAcc was strengthened by the anticipation of monetary gain. This modulation was also positively correlated with subjective pleasure ratings, especially anticipatory pleasure.

Consistent with results from previous fMRI studies (Knutson et al., 2000, 2001; Breiter et al., 2001; Zink et al., 2004), the NAcc is activated during anticipation of both reward and punishment in the present study. Earlier research in animals highlighted the role of the NAcc in reward anticipation, namely the “wanting” component of reward processing (Berridge and Robinson, 1998; Berridge, 2003). However, other animal studies had stressed the role of the NAcc in reward learning because local dopamine release was associated with novel stimuli and predictive cues indicating forthcoming reward or punishment (Schultz et al., 1997; Schultz, 2007). In human studies, the NAcc is conceptualized as a hedonic hotspot important in pleasure processing (Knutson and Greer, 2008; Kringelbach and Berridge, 2009). Knutson and Greer (2008) reviewed fMRI studies employing the MID task and suggested that the NAcc is involved in anticipating positive events and is associated with subjective pleasure and approaching behavior. Electrical stimulation of the NAcc in rodents and deep brain stimulation of the NAcc in humans both promote approaching behavior (Kringelbach and Berridge, 2009; Berridge and Kringelbach, 2013). Although a previous study had demonstrated that anticipation of reward rather than punishment activated the NAcc (Sabatinelli et al., 2007), activation of the NAcc had also been reported in anticipation of aversive stimuli in another study (Zink et al., 2004). Our findings lend support to the important role of NAcc in the processing of both salient information and pleasure during anticipation of rewards.

The role of the SN/VTA and the thalamus in reward processing is less controversial compared to the function of the NAcc discussed above. O’Doherty et al. (2002) found that the SN/VTA is activated during anticipation of glucose, whereas its role in anticipation of secondary rewards such as monetary stimuli is less clear. While Knutson et al. (2000, 2001) did not observe activation of the SN/VTA during anticipation of monetary stimuli, Breiter et al. (2001) identified activation in the VTA during anticipation of monetary rewards which was similar to our findings. Moreover, the VTA has been found to be activated in response to beautiful faces, which suggested that the midbrain may play an important role in positive social reward (Aharon et al., 2001). Activation of the SN/VTA to both monetary gain and loss identified in this study stresses the role of SN/VTA in reward processing.

Activation of the thalamus during anticipation of monetary gain and loss in the present study is consistent with results from a previous review (Knutson and Greer, 2008). While the thalamus is regarded as a center for information gathering and integration (Haber and Calzavara, 2009; Kohls et al., 2013), a previous study of electrical recording in rats suggested that the thalamus is also involved in retrospective and prospective coding to reward (Komura et al., 2001). In addition, the thalamus appears to be responsive to salient sensory information during the anticipation of incentives (Cho et al., 2013).

We also found a causal pathway from the SN/VTA to the NAcc during anticipation of monetary stimuli. To the best of our knowledge, few studies have investigated the causal relationship between the midbrain and the VS. A previous study on functional connectivity has reported that spontaneous activation of the NAcc correlated with reward-related brain circuits including the orbitofrontal cortex, the globus pallidus, the thalamus, the midbrain, the amygdala and the insular (Cauda et al., 2011). In addition, connectivity between the mesolimbic system and cortical areas appears to be altered in developmental conditions (Camara et al., 2009). The close relationship between mesolimbic connection and reward processing may be related to local dopaminergic metabolism. Schott et al. (2008) found that activation of the SN/VTA is associated with dopamine release in the NAcc. Moreover, Knutson and Gibbs (2007) identified that dopamine release in the NAcc activates postsynaptic D1 receptors which further induces activation in the NAcc during anticipation of reward. Both studies in functional connectivity and neurotransmitter stressed the role of mesolimbic connection in anticipation of positive events and pleasure, but the causal direction between the midbrain and the VS in this process is not clear. The causal connectivity from the SN/VTA to the NAcc identified in the present study is not only consistent with previous functional connectivity studies, but also provides new information regarding causal relationships in the mesolimbic pathway. Moreover, our findings support the role of the thalamus in integrating information from emotional, cognitive and motor cortical and subcortical areas to facilitate approaching and goal-directed behaviors (Haber and Calzavara, 2009; Haber and Knutson, 2010). In the best-fit model of our study, the SN/VTA projects an excitatory pathway to the thalamus, whereas the NAcc projects an inhibitory pathway to the thalamus. In another previous study which investigated brain circuits during anticipation of monetary incentives using DCM, Cho et al. (2013) found that the thalamus modulated the NAcc through the thalamus-to-NAcc and the thalamus-to-insula-to-NAcc connections which was different from our findings. We believe that the different findings may be related to the choice of the driven regions adopted. In the present study, the SN/VTA was chosen as the driven region, which is upstream to the dopaminergic complex in the NAcc (Schott et al., 2008; Duezel et al., 2009), whereas the thalamus was chosen as the driven region by Cho et al. (2013). However, a connection from the NAcc to the thalamus was also identified in Cho et al.’s (2013) study, which is similar to our findings. We found that both monetary ‘gain’ and ‘loss’ induced perturbation in all three connectivities in Model 8, namely the SN/VTA-to-thalamus connectivity, the SN/VTA-to-NAcc connectivity, and the NAcc-to-thalamus connectivity. In contrast to the study by Cho et al. (2013), which found different patterns of perturbation between monetary ‘gain’ and ‘loss’ in the thalamus-insula-NAcc circuit, our findings revealed a similar pattern regardless of valence in the SN/VTA-NAcc-thalamus network. The ROIs adopted and the driving ROI chosen may both cause the difference between the present and previous findings. The SN/VTA adopted here appears to play an elementary role in reward processing which may not be sensitive to the valence of the reward.

More interestingly, the modulation by monetary gain on the connectivity from the SN/VTA to the NAcc was correlated with anticipatory pleasure experience in the present study. The MID task and the TEPS are tools suggested by the RDoC for measuring anticipatory and consummatory pleasure in fMRI and behavioral paradigms, respectively, (NIMH, 2008), but few studies have investigated the underlying neural mechanism of the two instruments. People with schizophrenia spectrum disorders report dampened subjective anticipatory pleasure, while their consummatory pleasure is relatively preserved (Kring and Caponigro, 2010; Kring et al., 2011). In addition, previous studies have identified that people with schizophrenia showed reduced activation in the NAcc when anticipating monetary stimuli (Juckel et al., 2006, 2012; Walter et al., 2009). These results suggest that the two instruments seem to capture similar underlying neural mechanisms and our findings corroborated this in healthy adolescents. The connection from the SN/VTA to the NAcc in adolescents who reported higher anticipatory pleasure is more easily perturbed by positive events. This phenomenon may reflect inherent dopaminergic metabolism in the mesolimbic system and this hypothesis merits further research.

This study has several limitations. First, we only focused on the causal network in the mesolimbic system and did not investigate other important reward-related circuits such as the mesocortical system. The second limitation is that the adopted dopaminergic midbrain area is the SN rather than the VTA, but the boundary between the SN and the VTA in human is difficult to distinguish (Duezel et al., 2009). Furthermore, laterality was not taken into consideration in this study. We only focused on the right hemisphere which showed higher activation than the left. Finally, only nine models were tested in this study. Future identification of other relevant connectivity or models is needed.

## Conclusion

Anticipation of both monetary gain and loss activated the NAcc and other reward-related areas, namely the SN/VTA and the thalamus. The SN/VTA projects causal pathways to the NAcc and the thalamus, while the thalamus integrates information from the SN/VTA and the NAcc. Anticipatory pleasure appears to predict the susceptibility of causal connection from the SN/VTA to the NAcc to positive events. The present findings also lend support to the applicability of the RDoC in research.

## Conflict of Interest Statement

The authors declare that the research was conducted in the absence of any commercial or financial relationships that could be construed as a potential conflict of interest.

## References

[B1] Aharon I. Etcoff N. Ariely D. Chabris C. F. O’connor E. Breiter H. C. (2001). Beautiful faces have variable reward value: fMRI and behavioral evidence. *Neuron* 32 537–551. 10.1016/S0896-6273(01)00491-3 11709163

[B2] Alexander W. H. Brown J. W. (2010). Competition between learned reward and error outcome predictions in anterior cingulate cortex. *Neuroimage* 49 3210–3218. 10.1016/j.neuroimage.2009.11.065 19961940 PMC2818639

[B3] Berridge K. C. (2003). Pleasures of the brain. *Brain Cogn.* 52 106–128. 10.1016/S0278-2626(03)00014-9 12812810

[B4] Berridge K. C. Kringelbach M. L. (2013). Neuroscience of affect: brain mechanisms of pleasure and displeasure. *Curr. Opin. Neurobiol.* 23 294–303. 10.1016/j.conb.2013.01.017 23375169 PMC3644539

[B5] Berridge K. C. Robinson T. E. (1998). What is the role of dopamine in reward: hedonic impact, reward learning, or incentive salience? *Brain Res. Rev.* 28 309–369. 10.1016/s0165-0173(98)00019-8 9858756

[B6] Breiter H. C. Aharon I. Kahneman D. Dale A. Shizgal P. (2001). Functional imaging of neural responses to expectancy and experience of monetary gains and losses. *Neuron* 30 619–639. 10.1016/S0896-6273(01)00303-8 11395019

[B7] Camara E. Rodriguez-Fornells A. Ye Z. Munte T. F. (2009). Reward networks in the brain as captured by connectivity measures. *Front. Neurosci* 3:350–362. 10.3389/neuro.01.034.2009 20198152 PMC2796919

[B8] Cauda F. Cavanna A. E. D’agata F. Sacco K. Duca S. Geminiani G. C. (2011). Functional connectivity and coactivation of the nucleus accumbens: a combined functional connectivity and structure-based meta-analysis. *J. Cognit. Neurosci.* 23 2864–2877. 10.1162/jocn.2011.21624 21265603

[B9] Chan R. C. K. Li Z. Li K. Zeng Y. W. Xie W. Z. Yan C. (in press). Distinct processing of social and monetary rewards in late adolescents with trait anhedonia. *Neuropsychology*10.1037/neu0000233 26280299

[B10] Chan R. C. K. Shi Y. F. Lai M. K. Wang Y. N. Wang Y. Kring A. M. (2012). The temporal experience of pleasure scale (TEPS): exploration and confirmation of factor structure in a healthy Chinese sample. *PLoS ONE* 7:e35352 10.1371/journal.pone.0035352 PMC3329425 22530007

[B11] Cho Y. T. Fromm S. Guyer A. E. Detloff A. Pine D. S. Fudge J. L. (2013). Nucleus accumbens, thalamus and insula connectivity during incentive anticipation in typical adults and adolescents. *Neuroimage* 66 508–521. 10.1016/j.neuroimage.2012.10.013 23069809 PMC3949208

[B12] Cuthbert B. N. (2014). The RDoC framework: facilitating transition from ICD/DSM to dimensional approaches that integrate neuroscience and psychopathology. *World Psychiatry* 13 28–35. 10.1002/wps.20087 24497240 PMC3918011

[B13] Cuthbert B. N. Workgrp N. R. (2014). The RDoC framework: continuing commentary. *World Psychiatry* 13 196–197. 10.1002/wps.20140 24890074 PMC4102294

[B14] Duezel E. Bunzeck N. Guitart-Masip M. Wittmann B. Schott B. H. Tobler P. N. (2009). Functional imaging of the human dopaminergic midbrain. *Trends Neurosci.* 32 321–328. 10.1016/j.tins.2009.02.005 19446348

[B15] Friston K. J. Harrison L. Penny W. (2003). Dynamic causal modelling. *Neuroimage* 19 1273–1302. 10.1016/S1053-8119(03)00202-7 12948688

[B16] Gard D. E. Gard M. G. Kring A. M. John O. P. (2006). Anticipatory and consummatory components of the experience of pleasure: a scale development study. *J. Res. Pers.* 40 1086–1102. 10.1016/j.jrp.2005.11.001

[B17] Gonen T. Admon R. Podlipsky I. Hendler T. (2012). From animal model to human brain networking: dynamic causal modeling of motivational systems. *J. Neurosci.* 32 7218–7224. 10.1523/JNEUROSCI.6188-11.2012 22623666 PMC6622288

[B18] Gong Y. X. (1992). *Manual of Wechsler Adult Intelligence Scale-Chinese Version.* Changsha: Chinese Map Press.

[B19] Haber S. N. Calzavara R. (2009). The cortico-basal ganglia integrative network: the role of the thalamus. *Brain Res. Bull.* 78 69–74. 10.1016/j.brainresbull.2008.09.013 18950692 PMC4459637

[B20] Haber S. N. Knutson B. (2010). The reward circuit: linking primate anatomy and human imaging. *Neuropsychopharmacology* 35 4–26. 10.1038/npp.2009.129 19812543 PMC3055449

[B21] Horvitz J. C. (2000). Mesolimbocortical and nigrostriatal dopamine responses to salient non-reward events. *Neuroscience* 96 651–656. 10.1016/S0306-4522(00)00019-1 10727783

[B22] Juckel G. Friedel E. Koslowski M. Witthaus H. Oezguerdal S. Gudlowski Y. (2012). Ventral striatal activation during reward processing in subjects with ultra-high risk for schizophrenia. *Neuropsychobiology* 66 50–56. 10.1159/000337130 22797277

[B23] Juckel G. Schlagenhauf F. Koslowski M. Filonov D. Wuestenberg T. Villringer A. (2006). Dysfunction of ventral striatal reward prediction in schizophrenic patients treated with typical, not atypical, neuroleptics. *Psychopharmacology (Berl.)* 187 222–228. 10.1007/s00213-006-0405-4 16721614

[B24] Keuken M. C. Bazin P. L. Crown L. Hootsmans J. Laufer A. Müller-Axt C. (2014). Quantifying inter-individual anatomical variability in the subcortex using 7 T structural MRI. *Neuroimage* 94 40–46. 10.1016/j.neuroimage.2014.03.032 24650599

[B25] Knutson B. Fong G. W. Adams C. M. Varner J. L. Hommer D. (2001). Dissociation of reward anticipation and outcome with event-related fMRI. *Neuroreport* 12 3683–3687. 10.1097/00001756-200112040-00016 11726774

[B26] Knutson B. Gibbs S. E. B. (2007). Linking nucleus accumbens dopamine and blood oxygenation. *Psychopharmacology (Berl.)* 191 813–822. 10.1007/s00213-006-0686-7 17279377

[B27] Knutson B. Greer S. M. (2008). Anticipatory affect: neural correlates and consequences for choice. *Philos. Trans. R Soc. Lond. B Biol. Sci.* 363 3771–3786. 10.1098/rstb.2008.0155 18829428 PMC2607363

[B28] Knutson B. Westdorp A. Kaiser E. Hommer D. (2000). FMRI visualization of brain activity during a monetary incentive delay task. *Neuroimage* 12 20–27. 10.1006/nimg.2000.0593 10875899

[B29] Kohls G. Perino M. T. Taylor J. M. Madva E. N. Cayless S. J. Troiani V. (2013). The nucleus accumbens is involved in both the pursuit of social reward and the avoidance of social punishment. *Neuropsychologia* 51 2062–2069. 10.1016/j.neuropsychologia.2013.07.020 23911778 PMC3799969

[B30] Komura Y. Tamura R. Uwano T. Nishijo H. Kaga K. Ono T. (2001). Retrospective and prospective coding for predicted reward in the sensory thalamus. *Nature* 412 546–549. 10.1038/35087595 11484055

[B31] Kring A. M. Caponigro J. M. (2010). Emotion in schizophrenia: where feeling meets thinking. *Curr. Dir. Psychol. Sci.* 19 255–259. 10.1177/0963721410377599 22557707 PMC3340922

[B32] Kring A. M. Gard M. G. Gard D. E. (2011). Emotion deficits in schizophrenia: timing matters. *J. Abnorm. Psychol.* 120 79–87. 10.1037/a0021402 21058754

[B33] Kringelbach M. L. Berridge K. C. (2009). Towards a functional neuroanatomy of pleasure and happiness. *Trends Cogn. Sci.* 13 479–487. 10.1016/j.tics.2009.08.006 19782634 PMC2767390

[B34] Montaron M. F. Deniau J. M. Menetrey A. Glowinski J. Thierry A. M. (1996). Prefrontal cortex inputs of the nucleus accumbens-nigro-thalamic circuit. *Neuroscience* 71 371–382. 10.1016/0306-4522(95)00455-6 9053793

[B35] NIMH (2008). *Research Domain Criteria (RDoC)*. Available at: http://www.nimh.nih.gov/research-priorities/rdoc/index.shtml [accessed May 6, 2015].

[B36] O’Doherty J. P. Deichmann R. Critchley H. D. Dolan R. J. (2002). Neural responses during anticipation of a primary taste reward. *Neuron* 33 815–826. 10.1016/S0896-6273(02)00603-7 11879657

[B37] Sabatinelli D. Bradley M. M. Lang P. J. Costa V. D. Versace F. (2007). Pleasure rather than salience activates human nucleus accumbens and medial prefrontal cortex. *J. Neurophysiol.* 98 1374–1379. 10.1152/jn.00230.2007 17596422

[B38] Schott B. H. Minuzzi L. Krebs R. M. Elmenhorst D. Lang M. Winz O. H. (2008). Mesolimbic functional magnetic resonance imaging activations during reward anticipation correlate with reward-related ventral striatal dopamine release. *J. Neurosci.* 28 14311–14319. 10.1523/JNEUROSCI.2058-08.2008 19109512 PMC6671462

[B39] Schultz W. (2007). Behavioral dopamine signals. *Trends Neurosci.* 30 203–210. 10.1016/j.tins.2007.03.007 17400301

[B40] Schultz W. Dayan P. Montague P. R. (1997). A neural substrate of prediction and reward. *Science* 275 1593–1599. 10.1126/science.275.5306.1593 9054347

[B41] Shankman S. A. Katz A. C. Delizza A. A. Sarapas C. Gorka S. M. Campbell M. L. (2014). “Chapter 1: the different facets of anhedonia and their associations with different psychopathologies,” in: *Anhedonia: A Comprehensive Handbook*, ed. Ritsner M. S. (Dordrecht: Springer Science+Business Media).

[B42] Stephan K. E. Penny W. D. Moran R. J. Den Ouden H. E. M. Daunizeau J. Friston K. J. (2010). Ten simple rules for dynamic causal modeling. *Neuroimage* 49 3099–3109. 10.1016/j.neuroimage.2009.11.015 19914382 PMC2825373

[B43] Veldhuizen M. G. Douglas D. Aschenbrenner K. Gitelman D. R. Small D. M. (2011). The anterior insular cortex represents breaches of taste identity expectation. *J. Neurosci.* 31 14735–14744. 10.1523/JNEUROSCI.1502-11.2011 21994389 PMC3221736

[B44] Walter H. Kammerer H. Frasch K. Spitzer M. Abler B. (2009). Altered reward functions in patients on atypical antipsychotic medication in line with the revised dopamine hypothesis of schizophrenia. *Psychopharmacology (Berl.)* 206 121–132. 10.1007/s00213-009-1586-4 19521678

[B45] Yu H. Zhou Z. Zhou X. (2013). The amygdalostriatal and corticostriatal effective connectivity in anticipation and evaluation of facial attractiveness. *Brain Cogn.* 82 291–300. 10.1016/j.bandc.2013.04.011 23774678

[B46] Zink C. F. Pagnoni G. Martin-Skurski M. E. Chappelow J. C. Berns G. S. (2004). Human striatal responses to monetary reward depend on saliency. *Neuron* 42 509–517. 10.1016/S0896-6273(04)00183-7 15134646

