# Supplementary material for: Anticipatory pleasure predicts effective connectivity in the mesolimbic system
Source: Front Behav Neurosci. 2015 Aug 12;9:217. doi: 10.3389/fnbeh.2015.00217 (PMC4532926; doi:10.3389/fnbeh.2015.00217)
Supplement: Supplementary file 1 [file Table_1.DOCX]

Supplementary Table 1. Behavioral performance on MID.

|  | Neutral cue | | Loss cue | | Gain cue | | Average | | F(df) | *p* |
| --- | --- | --- | --- | --- | --- | --- | --- | --- | --- | --- |
|  | Mean | SD | Mean | SD | Mean | SD | Mean | SD |  |  |
| Reaction Time | 234.16 | 37.37 | 238.68 | 25.07 | 241.21 | 24.97 | 238.45 | 24.82 | 0.75(2) | 0.474 |
| Hit rate | 58.85% | 6.46% | 61.35% | 7.68% | 58.27% | 9.01% | 59.49% | 4.73% | 2.29(2) | 0.104 |
| Final earnings | / | / | / | / | / | / | 22.10 | 5.56 | / | / |

Note: Significant difference was not found between the neutral cue, the loss cue and the gain cue on the reaction time and the hit rate.
